# Supplementary material for: Rhizospheric Bacillus spp. Exhibit Miticidal Efficacy against Oligonychus coffeae (Acari: Tetranychidae) of Tea
Source: Microorganisms. 2023 Nov 2;11(11):2691. doi: 10.3390/microorganisms11112691 (PMC10673310; doi:10.3390/microorganisms11112691)
Supplement: Supplementary file 1 [file microorganisms-11-02691-s001.zip › microorganisms-2469673-supplementary.pdf]

SUPPLEMENTARY DATA

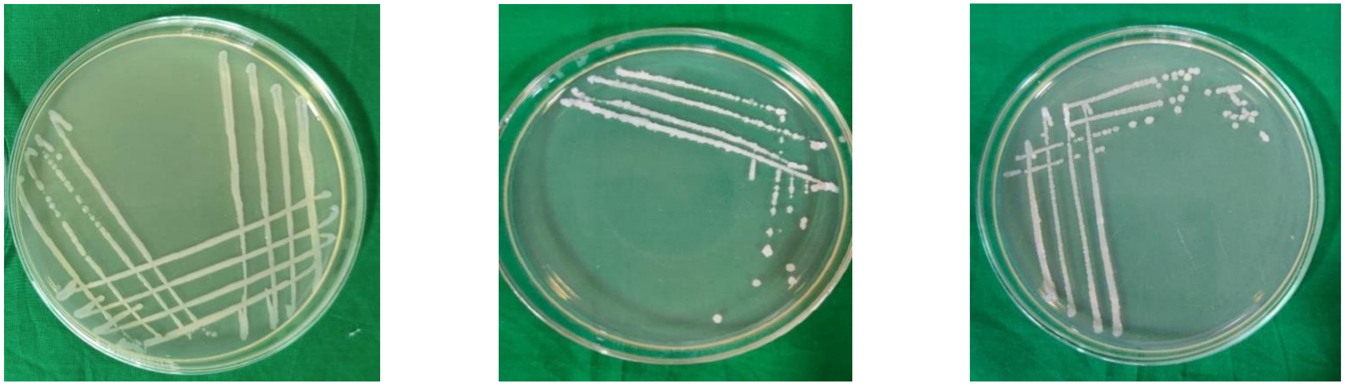

Figure S1. Different *Bacillus* spp. used for the study
(A) *Bacillus amyloliquefaciens* BAC1 (ON392425); (B) *B. subtilis* LB22 (ON386193); (C) *B. velezensis* AB22 (ON209629)

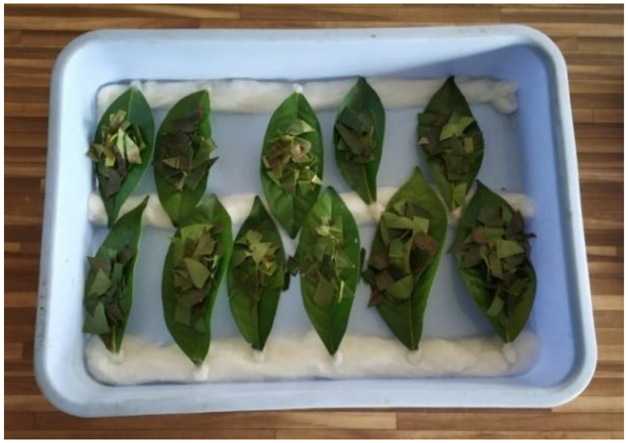

Figure S2. Rearing of Red Spider Mite (RSM) in a plastic tray

Table S1 Adulticidal activity of different *Bacillus* spp. on RSM

Treatment	Adulticidal activity of RSM at different hours after treatment (HAT)															
	24 hrs				48 hrs				72 hrs				96 hrs			
	1x 10^7	1x 10^8	1x 10^9	Mean	1x 10^7	1x 10^8	1x 10^9	Mean	1x 10^7	1x 10^8	1x 10^9	Mean	1x 10^7	1x 10^8	1x 10^9	Mean
T1: *Bacillus velenzensis* AB22	65.00<br>(53.73)	71.67<br>(57.80)	85.00<br>(67.21)	73.89<br>(59.21)	76.67<br>(61.07)	83.33<br>(65.88)	91.67<br>(73.15)	83.89<br>(66.27)	86.67<br>(68.53)	90.00<br>(71.57)	100.00<br>(90.00)	92.23<br>(73.78)	93.33<br>(75.00)	96.67<br>(79.37)	100.00<br>(90.00)	96.67<br>(79.37)
T2: *B. amyloliquefaciens* BAC1	56.67<br>(48.79)	68.33<br>(55.73)	76.67<br>(61.07)	67.22<br>(55.06)	70.00<br>(56.79)	78.33<br>(62.24)	83.33<br>(65.88)	77.22<br>(61.48)	80.00<br>(63.43)	86.67<br>(68.53)	91.67<br>(73.15)	86.11<br>(68.11)	88.33<br>(70.00)	91.67<br>(73.15)	100.00<br>(90.00)	93.33<br>(75.00)
T3: *B. subtilis* LB22	46.67<br>(43.05)	55.00<br>(47.87)	63.33<br>(52.71)	55.00<br>(47.87)	65.00<br>(53.73)	71.67<br>(57.80)	78.33<br>(62.24)	71.67<br>(57.80)	76.67<br>(61.07)	81.67<br>(64.60)	86.67<br>(68.53)	81.67<br>(64.60)	81.67<br>(64.60)	88.33<br>(70.00)	93.33<br>(75.00)	87.77<br>(69.47)
T4: Control	0.00<br>(0.00)	0.00<br>(0.00)	0.00<br>(0.00)	0.00<br>(0.00)	0.00<br>(0.00)	0.00<br>(0.00)	0.00<br>(0.00)	0.00<br>(0.00)	0.00<br>(0.00)	0.00<br>(0.00)	0.00<br>(0.00)	0.00<br>(0.00)	0.00<br>(0.00)	0.00<br>(0.00)	0.00<br>(0.00)	0.00<br>(0.00)
Mean	52.91<br>(46.66)	48.75<br>(44.26)	56.25<br>(48.56)		52.91<br>(46.65)	58.33<br>(49.78)	63.33<br>(52.71)		60.83<br>(51.24)	64.58<br>(53.43)	69.58<br>(56.48)		65.83<br>(54.21)	69.16<br>(56.23)	73.33<br>(58.89)	
	CD (P= 0.05)		SED		CD (P= 0.05)		SED		CD (P= 0.05)		SED		CD (P= 0.05)		SED	
Treatment (T)	0.400		0.200		0.309		0.154		0.286		0.143		0.232		0.116	
Conc. (C)	0.347		0.173		0.268		0.134		0.248		0.124		0.201		0.1	
Interaction (TxC)	0.693		0.346		0.535		0.267		0.496		0.247		0.402		0.201	

Figures in parentheses square root transformed values

Table S2. Ovicidal activity of different *Bacillus* spp. on RSM

Treatment	Ovicidal activity of RSM at 14 days after spray (DAS)			
	1x 10^7	1x 10^8	1x 10^9	Mean
T1: *Bacillus velenzensis* AB22	87.22<br>(69.06)	92.78<br>(74.41)	100.00<br>(90.00)	93.34<br>(75.00)
T2: *B. amyloliquefaciens* BAC1	84.44<br>(66.77)	87.22<br>(69.06)	92.22<br>(73.81)	87.96<br>(69.64)
T3: *B. subtilis* LB22	77.78<br>(61.87)	81.67<br>(64.65)	86.67<br>(68.58)	82.03<br>(64.90)
T4: Control	0.00<br>(0.00)	0.00<br>(0.00)	0.00<br>(0.00)	0.00<br>(0.00)
Mean	62.36<br>(52.12)	65.41<br>(53.97)	69.72<br>(56.60)	
	CD (P= 0.05)		SED	
Treatment (T)	0.707		0.353	
Concentration (C)	0.613		0.306	
Interaction (TXC)	1.225		0.611	
